# PAPC and the Wnt5a/Ror2 pathway control the invagination of the otic placode in Xenopus

**DOI:** 10.1186/1471-213X-11-36

**Published:** 2011-06-10

**Authors:** Barbara Jung, Almut Köhler, Alexandra Schambony, Doris Wedlich

**Affiliations:** 1 Karlsruhe Institute of Technology, Campus South, Zoological Institute, Department of Cell and Developmental Biology, Fritz-Haber-Weg 4, Karlsruhe, 76131, Germany; 2 Current Address: University of Freiburg, Institute of Biology I, Department of Developmental Biology, Hauptstr. 1, Freiburg, 79104, Germany; 3 Current Address: University of Erlangen-Nuernberg, Biology Department, Developmental Biology, Staudtstr. 5, Erlangen, 91058, Germany

## Abstract

**Background:**

Paraxial protocadherin (PAPC) plays a crucial role in morphogenetic movements during gastrulation and somitogenesis in mouse, zebrafish and Xenopus. PAPC influences cell-cell adhesion mediated by C-Cadherin. A putative direct adhesion activity of PAPC is discussed. PAPC also promotes cell elongation, tissue separation and coordinates cell mass movements. In these processes the signaling function of PAPC in activating RhoA/JNK and supporting Wnt-11/PCP by binding to frizzled 7 (fz7) is important.

**Results:**

Here we demonstrate by loss of function experiments in Xenopus embryos that PAPC regulates another type of morphogenetic movement, the invagination of the ear placode. Knockdown of PAPC by antisense morpholinos results in deformation of the otic vesicle without altering otocyst marker expression. Depletion of PAPC could be rescued by full-length PAPC, constitutive active RhoA and by the closely related PCNS but not by classical cadherins. Also the cytoplasmic deletion mutant M-PAPC, which influences cell adhesion, does not rescue the PAPC knockdown. Interestingly, depletion of Wnt5a or Ror2 which are also expressed in the otocyst phenocopies the PAPC morphant phenotype.

**Conclusions:**

PAPC signaling via RhoA and Wnt5a/Ror2 activity are required to keep cells aligned in apical-basal orientation during invagination of the ear placode. Since neither the cytoplasmic deletion mutant M-PAPC nor a classical cadherin is able to rescue loss of PAPC we suggest that the signaling function of the protocadherin rather than its role as modulator of cell-cell adhesion is required during invagination of the ear placode.

## Background

Paraxial protocadherin PAPC stands out among the cadherin superfamily members by its binding partners, signaling activity and specific expression pattern. It is highly conserved among vertebrates and functional homology has been observed in mouse, zebrafish and Xenopus. The human ortholog is named PCDH8 (protocadherin 8) and localized on chromosome 13 [1]. PCDH8 is found repressed in many breast tumors by mutations or epigenetic silencing, which results in up-regulation of proliferation and invasion [2]. Arcadlin, the rat ortholog of PAPC, is expressed during brain development in the hippocampus, the auditory, the visual and the limbic systems [3]. Arcadlin binds to N-Cadherin and promotes its endocytosis thereby controlling the dentritic spine number [4].

In Xenopus, xPAPC was identified in a screen for Spemann organizer genes [5]. Functional studies revealed its significance in mass cell movements during gastrulation. xPAPC is essential to keep the involuting mesoderm separated from the overlying neuroectoderm so that the Brachet's cleft is formed [6]. In addition xPAPC coordinates the mesoderm cells to converge towards the dorsal midline [7]. Both activities depend on the ability of xPAPC to activate the small GTPase RhoA. The latter might be supported by xANR5, a cytoplasmic binding partner of xPAPC, which regulates tissue separation and cell protrusion formation in gastrulation [8]. The extracellular domain of xPAPC was shown to decrease C-Cadherin mediated cell-cell adhesion by a mechanism, which is not yet understood. This activity of xPAPC does not depend on frizzled-7 (xFz7) [9].

PAPC is also required for the segmentation of somites. The latter has been reported for Xenopus, mouse and zebrafish [10-12]. Zebrafish PAPC is under control of the T-box factor Tbx16; its mutant spadetail shows reduced zPAPC expression. The spadetail gastrulation phenotype resembles the phenotype observed after expression of dominant-negative zPAPC [13,14]. In Xenopus Medina et al. [6] have demonstrated that xPAPC is linked to the non-canonical Wnt-PCP pathway because its extracellular domain interacts with xFz7. In addition, xPAPC antagonizes the inhibitory influence of sprouty on Wnt-PCP signaling by binding to it [15]. Apart from being regulated by activin/BVg1, nodal and β-catenin [6,16,17], xPAPC expression is under control of the non-canonical Wnt5a/Ror2 pathway [18]. Importantly, only depletion of Wnt5a or Ror2 mimics the xPAPC-loss-of-function phenotype in gastrulation movement while depletion of the other regulators strongly affects mesoderm induction and patterning.

Apart from the Spemann organizer and somatic mesoderm xPAPC is expressed in the developing inner ear [19]. The development of the inner ear in vertebrates involves morphogenetic movements, which include invagination of the otic placode, delamination of the neuroblasts and reshaping the otocyst into otic structures like semicircular canals. Important signaling pathways involved in the induction of the otic placode have been identified in mouse, chicken, zebrafish and Xenopus [20,21]. Wnt-PCP signaling and protocadherins are required in the late period of stereocilia formation during differentiation of the organ Corti (reviewed in [22,23]). However, little is known about the role of protocadherins and non-canonical Wnt-signaling in the morphogenetic movements during otocyst and neuroblast formation.

Here we demonstrate by antisense morpholino injections that xPAPC signaling rather than its adhesive function is required for proper invagination of the otic placodal epithelium. Constitutive active RhoA and PCNS, the closest relative to xPAPC, rescued depletion of xPAPC whereas classical cadherins and M-PAPC did not. Interestingly, depletion of Wnt5a and Ror2, both, phenocopied xPAPC loss of function.

## Results

### XPAPC is expressed with Wnt5a, Ror2 and xFz7 in the otic anlage

**Figure 1:**
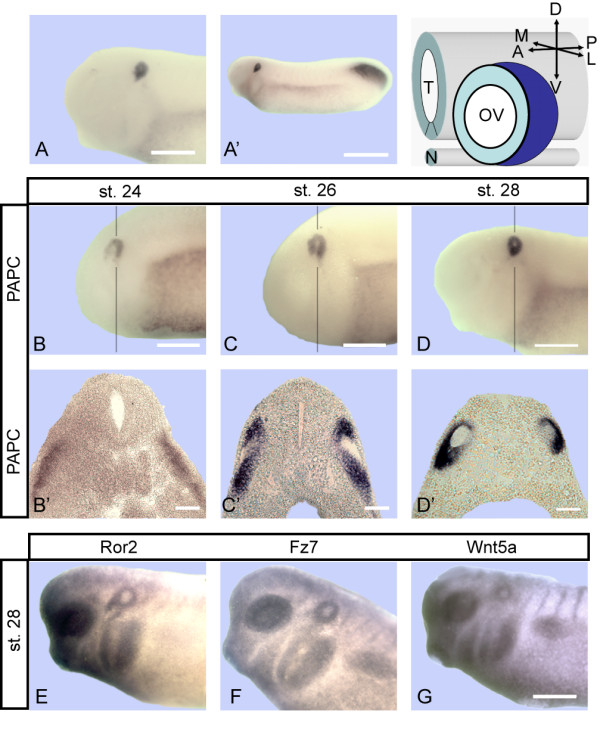
**Expression of xPAPC, Ror2, xFz7 and Wnt5a in the otic vesicle**. **(A, A') **ISH for xPAPC expression at tailbud stage (stage 28). **(A) **Enlarged head shows a strong signal in the otic region. **(A') **Additional expression domain at the tail organizer. **(B, C, D) **xPAPC expression in the otic vesicle at stages 24, 26 and 28. Vertical line indicates level of section. The corresponding transversal sections **(B', C', D') **reveal a strong restriction to the lateral epithelium. The scheme shows axis orientation in the otic vesicle in regard to the axial organs neural tube and notochord. **(E, F, G) **Otic expression of Ror2 (E), xFz7 (F) and Wnt5a (G) in a stage 28 embryo. A, anterior; D, dorsal; L, lateral; M, medial; N, notochord; OV, otic vesicle; P, posterior; T, neural tube; V, ventral; Scale bar (A, C, F) 500 μm, (A') 600 μm, (C') 100 μm.

During gastrulation and neurulation xPAPC is expressed in Spemann's organizer and in presomitic mesoderm [5] whereas in later development its expression is restricted to the otic vesicle and the tail organizer (Figure 1A, A'). At stage 24 and 26 the otocyst appeared "open" because xPAPC transcripts are missing in the ventrolateral compartment (Figure 1B, B', C, C', for axis orientation see scheme). At stage 28, the in situ hybridization signal (ISH) for xPAPC formed a donut-like shape with a light spot in the centre, which marks the inner cavity. xPAPC expression has shifted laterally, while the signal has faded in the medial epithelium compartment (Figure 1D, D').

xPAPC is regulated by the non-canonical Wnt5a/Ror2 pathway, which cooperates with the Wnt/PCP pathway [18]. Therefore we tested whether key components of these pathways are also expressed in the otic anlage. Wnt5a, Ror2 and xFz7 were found expressed in the otic vesicle but also in the eye and neural crest (Figure 1E, F, G).

### Knockdown of xPAPC results in malformation of the otocyst epithelium altering localization of adhesion proteins and aPKC

**Figure 2:**
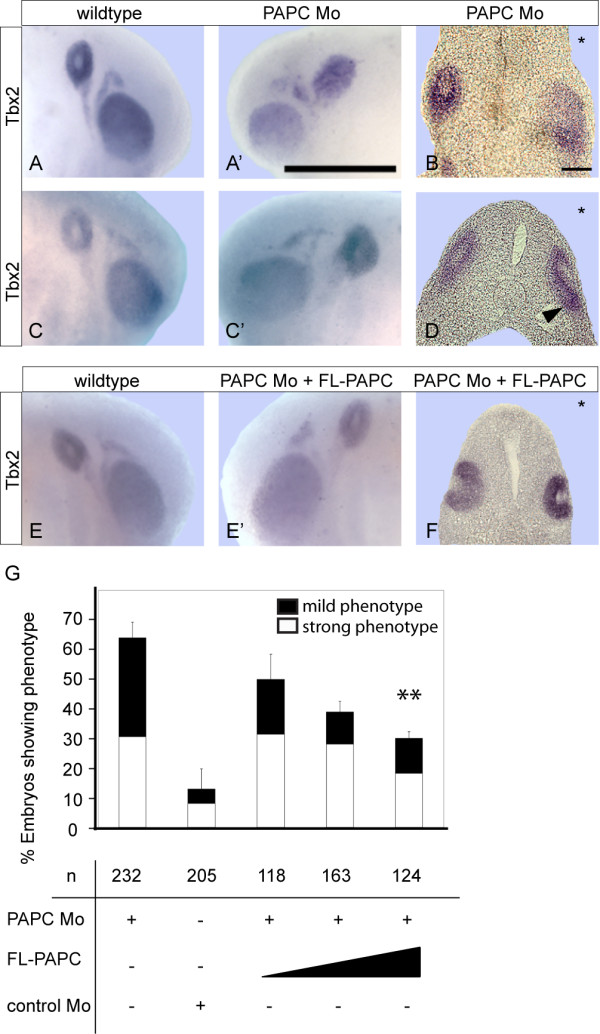
**Knockdown of xPAPC leads to otocyst deformation**. **(A, A', B, C, C', D) **ISH for Tbx2 shows an altered expression pattern at the injected side **(A', C') **compared to the wildtype side **(A, C)**. A severe phenotype is shown in **(A, A', B)**, a mild phenotype in **(C, C', D)**. Corresponding transversal sections are presented in **(B, D)**. Note the infolding epithelium at the injected side, arrowhead. **(E, E', F) **Rescue by coinjection of 500 pg FL-PAPC RNA, **(E) **wildtype, **(E') **PAPC Mo coinjected with full-length PAPC, **(F) **corresponding transversal section. **(G) **Statistical evaluation of xPAPC depletion and rescue experiments at stage 26. The embryos are single side injected and phenotypes were examined via Tbx2 ISH. FL-PAPC RNA was coinjected in increasing amounts (100 pg, 250 pg, 500 pg), also a control Morpholino was injected to eliminate the possibility of unspecific effects. The data are normalized relating to uninjected wildtype embryos. FL, full-length; Mo, antisense morpholino; n, number of embryos. Injected side is indicated by asterisks in transversal sections. Scale bar (A') 500 μm, (B) 100 μm. ** = p < 0,01

Next we studied the function of xPAPC during inner ear development by antisense morpholino (PAPC Mo) injections into one blastomere of two-cell stage embryos. The efficiency of PAPC Mo was shown previously [7]. Depletion of xPAPC resulted in deformation of the ear vesicle, which was monitored by ISH for Tbx2. On the PAPC Mo injected side otocysts appeared flattened and the Tbx2 signal was slightly enlarged (Figure 2A, A', B and 2C, C', D). Severe phenotypes revealed a loss of the otocyst cavity, which was visible from outside by the lack of the unstained spot in the centre (Figure 2A'). Cross-sections of strong phenotypes verified that no cavity was formed (Figure 2B). Weaker phenotypes exhibited a less pronounced cavity and the vesicle epithelium appeared broader with a more diffuse margin (Figure 2C, C'). In cross-sections a cavity was partially formed while the broadening of the epithelium was caused by irregular upfolding (Figure 2D, arrowhead). As indicated in the statistical evaluation, the ratio between strong and weak phenotype among the morphants was nearly 1:1 (Figure 2G). The specificity of the xPAPC Mo phenotype was confirmed by injection of the control morpholino (Control Mo), which did not lead to abnormalities of the ear vesicle's shape. Furthermore, the defects in the otocyst morphology caused by xPAPC depletion could be rescued by FL-PAPC RNA co-injection (Figure 2E, E', F, G).

**Figure 3:**
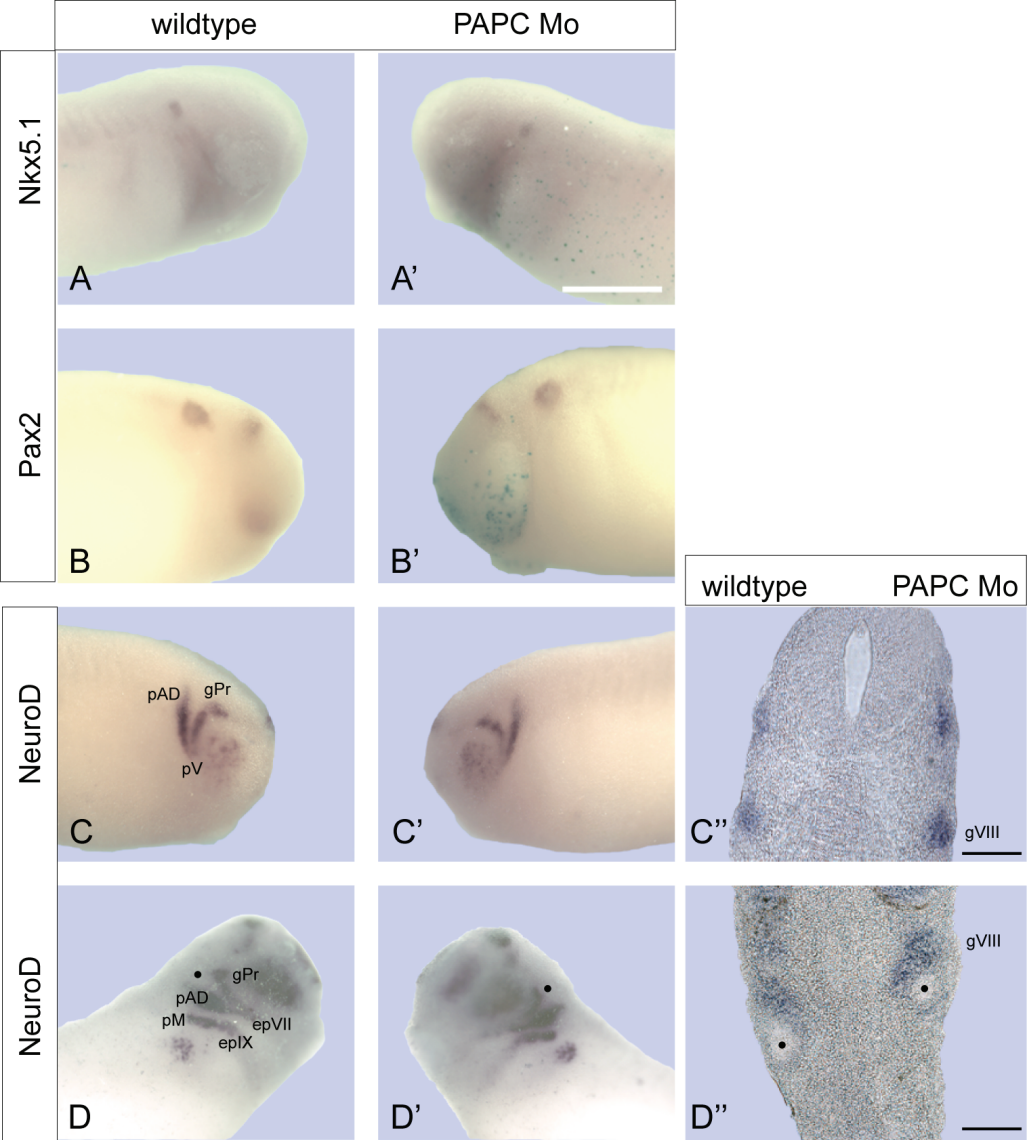
**xPAPC depletion does not affect patterning and innervation events**. xPAPC antisense morpholinos were single side injected to examine possible effects in patterning (**A, A', B, B') **or innervation **(C, C', D, D'). (A, A', B, B') **ISH at stage 26 for the anterior otic marker Nkx5.1, n = 15 **(A, A') **and the ventral marker Pax2, n = 31 **(B, B')**. xPAPC depletion shows no impact. **(C, C', D, D') **ISH for NeuroD at stage 26, n = 21 **(C, C', C'') **and stage 28, n = 8 **(D, D', D'')**. NeuroD expression is unaffected from xPAPC knockdown. Black dot marks the otic vesicle. epIX, epVII = epibranchial placodes; gVIII = statoacoustic ganglion; gPr = profundal placode; pAD = anterodorsal lateral line placode; pM = middle lateral line placode. Scale bar 500 μm (A-C', D-D'), 100 μm (C'', D'').

Since xPAPC depletion did not affect otic placode induction, seen by Tbx2 (Figure 2), Pax8 (additional file 1) and Sox10 (data not shown) expression, we next asked whether compartmentalization of the ear vesicle was affected. Therefore, we performed ISH for Pax2 and Nkx5.1, which are early markers for the dorsal and the anterior domain, respectively [24,25]. The expression domains of these markers remained unaltered upon PAPC Mo injections (Figure 3A, A', B, B'). Neuroblasts also derive from the otic placode. To prove whether neuroblast development was affected by xPAPC depletion ISH for NeuroD was performed which labels the peripheral nerve system including the neuroblasts derivatives [26]. As seen in Figure (3C, C', C'', D, D', D'') the cranial nerves and ganglia develop normally in xPAPC depleted embryos. We also investigated Runx1 and Tbx1 expression in tailbud stage 37 by ISH and the peripheral nervous system in tadpoles (stage 47) by immunostaining with an antibody against neurofilament (3A10). As seen in additional file 1 PAPC Mo injection revealed no phenotype. Thus, neither induction nor compartmentalization or differentiation of the otic tissues was impaired by loss of xPAPC. Therefore we assumed that the morphogenetic movement but not gene expression is disturbed in inner ear development of morphants.

**Figure 4:**
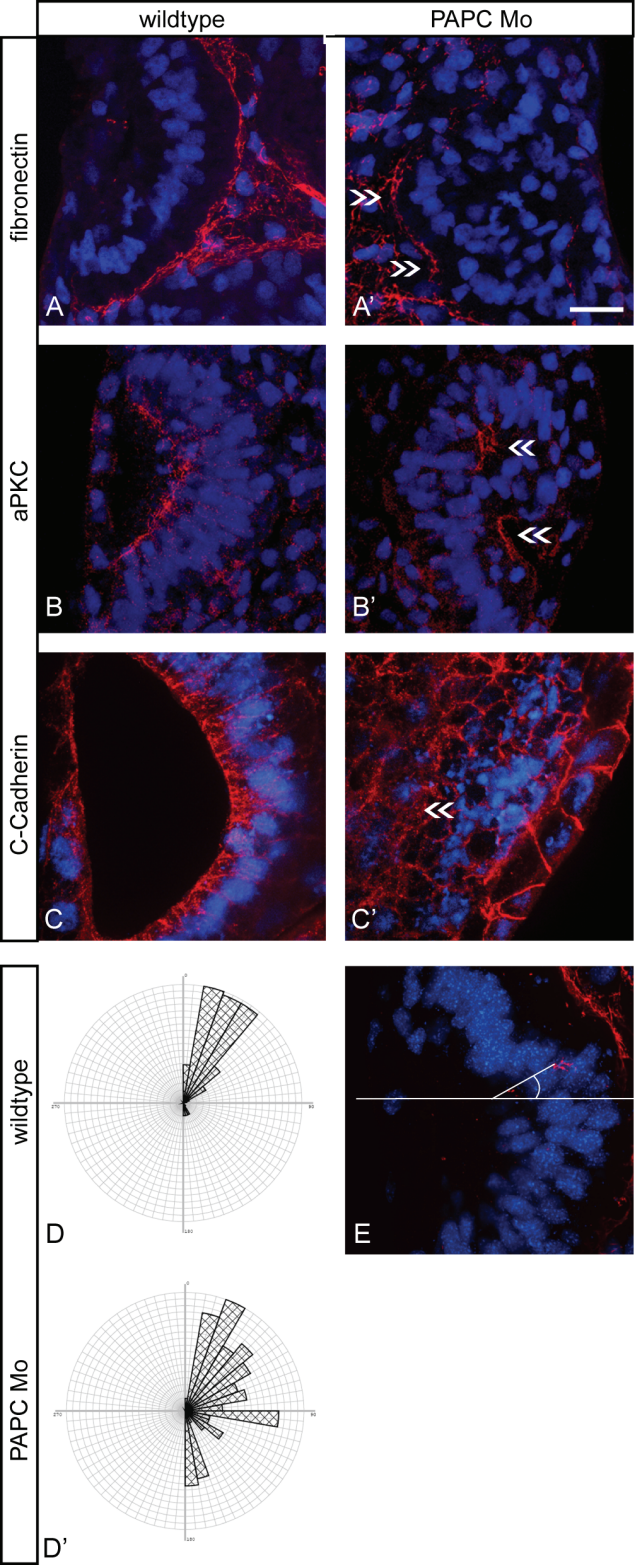
**Immunostaining of otic epithelia**. **(A, A', B, B', C, C') **ISH for Tbx2 was performed at stage 26. Embryos were then sliced and immunostained for different epithelial markers, like **(A, A') **fibronectin (n = 23), **(B, B') **aPKC (n = 19) and **(C, C') **C-Cadherin (n = 23) in red. Nuclei were stained with DAPI in blue. The otocyst region could be identified in the sections by Tbx2 expression. **(A', B', C') **Injected otocysts show epithelial disorganization indicated through inaccurate nuclei alignment and displaced epithelial markers (arrows). **(D, D') **Rose diagram to highlight the orientation of cells in wildtype **(D) **compared to morphant **(D') **otocyst region. Wildtype otocysts show cells orientated mostly in angles between 0 and 45°while morphant otocysts display angles between 0 and 180°. **(E) **Demonstration of the angle measurements: orientation of DAPI stained nuclei in relation to a horizontal median through the otocyst. Scale bar 20 μm.

For better characterization of the phenotype transverse sections of the ear vesicle were immunostained for epithelial markers. Staining for fibronectin, which labels the basal lamina of the epithelium in control otocysts (Figure 4A) revealed, that a fibronectin meshwork instead of a laminar structure was formed upon xPAPC depletion (Figure 4A', arrows). DAPI nuclear staining showed failures in the correct orientation of epithelial cells. We quantified this defect in epithelial cell alignment by measuring the angles of the nuclei to the mediolateral axis in transverse sections (E). The values were displayed in rose diagrams (D, D'). On the control side the cell nuclei of the otic vesicle epithelium of proper columnar morphology were arranged in angles between 0 - 45°(Figure 4D) to the mediolateral axis of the vesicle. Epithelial cells on the injected side, however, were found more randomly orientated in angles between 0 and 180°(Figure 4D'). This fact points to an incorrect upfolding of the otic epithelium (Figure 4A'). The latter was further confirmed by immunostaining for aPKC, which marks the apical surface of the otocyst epithelium in controls (Figure 4B). In PAPC depleted otocysts aPKC was only found in small subsets of cells demonstrating an incorrect orientation of the apical cell surfaces (Figure 4B'). C-Cadherin was also present in malformed otocysts but irregularly distributed (Figure 4C, C'). As already seen by *Tbx *ISH (Figure 2A', B) the immunostaining of sections also revealed that no cavity is formed during ear placode invagination (Figure 4A', B', C'). Comparable results were obtained for E-Cadherin, XB-Cadherin and β-Catenin, which all were found present in atypically arranged otocyst epithelia after PAPC Mo injections. Thus, lack of xPAPC resulted in the failure of the otocyst cells to align in apical-basal orientation and to build up a proper epithelium.

### xPAPC function in inner ear development can be replaced by the protocadherin PCNS but not by classical cadherins

**Figure 5:**
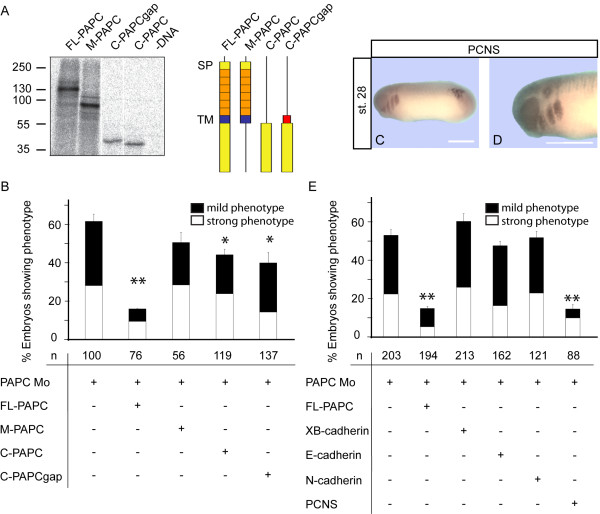
**Functional analysis of xPAPC during otic development**. **(A, B) **Coinjection of different functional xPAPC domains with xPAPC Mo. **(A) **Constructs that were coinjected in **(B)**, comprise of six characteristic extracellular cadherin domains (orange), transmembrane domain (blue), gap43 membrane anchor (red) or cytoplasmic part (yellow). All used constructs were tested for successful expression in a radioactive TNT assay. **(B) **500 pg RNA were coinjected with xPAPC Mo into one blastomere of 2-cell stage. At stage 26 the embryos were examined via ISH for Tbx2. The data are normalized relating to uninjected wildtype embryos. Only coinjection of FL-PAPC showed reconstitution, C-PAPC and C-PAPCgap partially rescued. **(C, D) **ISH for PCNS at tailbud stage (stage 28). **(D) **Enlarged head shows a strong signal in the otic region, as well as in branchial arches. **(E) **Reconstitution analysis of xPAPC Mo through coinjection of classical cadherins or PCNS. 500 pg of RNA were coinjected with xPAPC Mo into one blastomere of 2-cell stage. At stage 26 the embryos were examined via Tbx2 ISH. The data are normalized relating to uninjected wildtype embryos. xPAPC function in inner ear development could be replaced by the protocadherin PCNS but not by classical cadherins. C, cytoplasmic; FL, full-length; M, membrane; Mo, antisense morpholino; n, number of embryos; SP, signal peptide; TM, transmembrane domain. Scale bar 500 μm. * = p < 0,05; ** = p < 0,01

xPAPC has been described to possess adhesive properties [5] but also to modulate C-Cadherin mediated cell-adhesion [9]. Therefore we performed reconstitution experiments with the membrane anchored extracellular (M-PAPC) and two versions of the cytoplasmic domain, a soluble cytosolic form (C-PAPC) and a membrane anchored cytosolic form (C-PAPCgap). All truncated proteins were efficiently synthesized (Figure 5A). M-PAPC did not rescue the xPAPC Mo phenotype while the FL-PAPC did so (Figure 5B, see also Figure 2E' and 2F). A partial rescue was observed with the extracellular deletion mutants C-PAPC and C-PAPCgap. We also investigated the rescue ability of the classical cadherins which are known to be expressed during inner ear development. None of the so far tested classical cadherins showed rescue abilities (Figure 5E). Instead, PCNS, another protocadherin closely related to xPAPC [27] and also expressed in the otic vesicle (Figure 5C, D), abolished the xPAPC knockdown phenotype in a similar range as FL-PAPC (Figure 5B, E). These results exclude a role of xPAPC in mediating cell-cell adhesion otherwise a classical cadherin would be able to rescue the otocyst phenotype. Also a function as modulator of C-Cadherin mediated adhesion can be ruled out because M-PAPC, which behaves as full-length PAPC in this process [9] did not restore knockdown of xPAPC in early inner ear development (Figure 5C).

### xPAPC depletion is rescued by RhoA but not by disheveled

**Figure 6:**
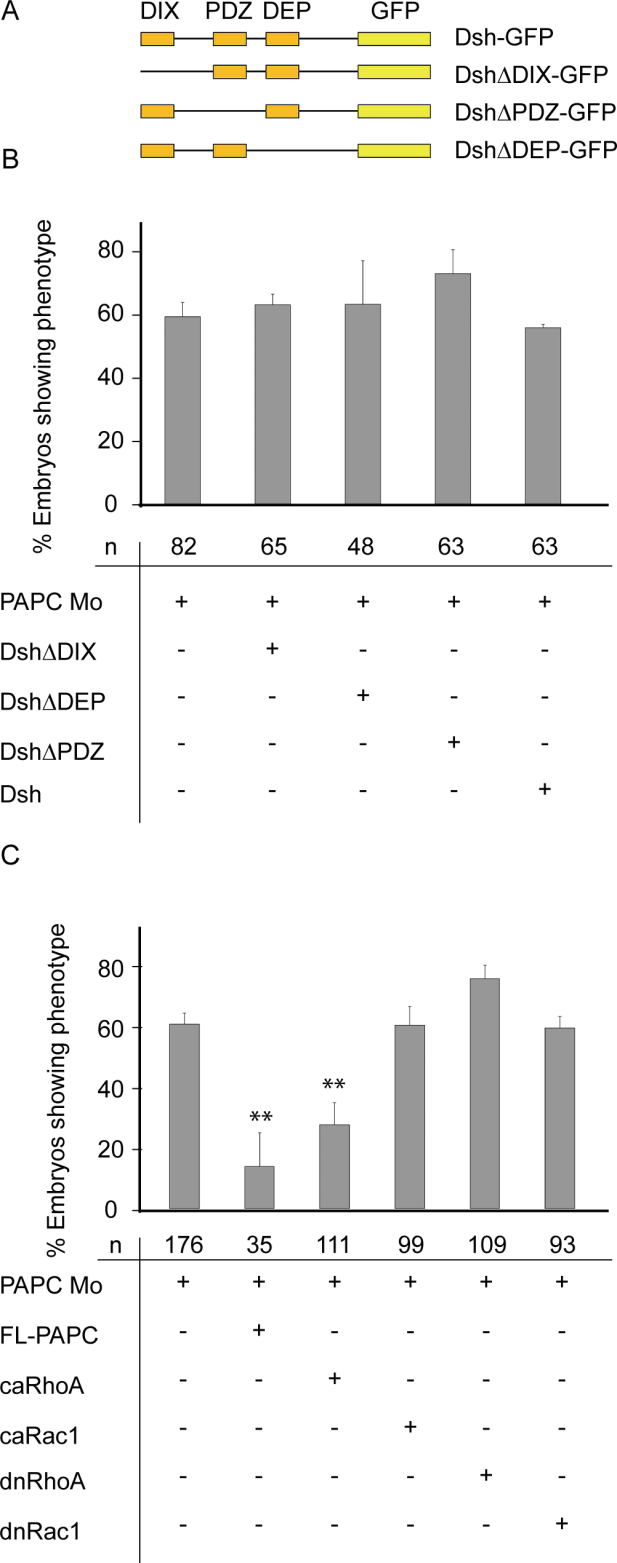
**xPAPC depletion is rescued by ca RhoA but not by disheveled**. **(A, B) **Coinjection of different disheveled constructs, shown in **(A). (B) **500 pg RNA were single side coinjected with xPAPC Mo at 16-cell stage in every one of the two adjacent ventral and dorsal cells in the animal part. At stage 26 the embryos were examined via Tbx2 ISH. The data are normalized relating to uninjected wildtype embryos. None of the constructs showed a reconstitution, so neither the Dsh-coupled PCP, nor canonical Wnt signaling pathway is involved in PAPC function. **(C) **Small GTPases Rac1 and RhoA were coinjected with xPAPC Mo as dominant negative (dn) or constitutive active (ca) constructs. The injection was carried out as described in (B). To further reduce secondary effects the injection was carried out with DNA. Reconstitution can be achieved by coinjection of 5 pg ca RhoA. Ca, constitutive active; Dn, dominant negative; Dsh, disheveled; FL, full length; Mo, antisense morpholino; n, number of embryos. ** = p < 0,01.

xPAPC also mediates alignment of mesodermal cells during convergent extension (CE) in gastrulation thereby driving mass cell migration towards the dorsal midline [7]. In this process xPAPC function depends on activation of RhoA while the simultaneously active Wnt-PCP signaling pathway requires RhoA and Rac1 activity [28]. Since xPAPC is able to bind to xFz7 [6], the receptor of Wnt-PCP cascade, an interference between the protocadherin and the cell polarity inducing pathway seems obvious. This prompted us to test the rescue capacities of different disheveled constructs and the small GTPases RhoA and Rac1. Disheveled (dsh) consists of three important domains, the DIX-domain required for canonical Wnt-signaling, the DEP-domain for Wnt-PCP signaling and the PDZ-domain for interaction with frizzled [29]. Dsh constructs lacking one of these domains are used in rescue experiments to distinguish between different Wnt-signaling pathways. As shown in figure (6A, B) none of the disheveled constructs was able to abrogate the otocyst abnormalities caused by xPAPC depletion. This indicates that Wnt-PCP and canonical β-catenin/Wnt signaling do not contribute to the phenotype. The latter was also confirmed by xFz7 knockdown experiments, which did not affect otocyst development (data not shown). Instead, activation of RhoA by xPAPC independent of the Wnt-PCP signaling is required for proper otocyst development. Reconstitution experiments with constitutive active (ca) RhoA but not with dominant-negative (dn) RhoA, dn Rac1 or ca Rac1 resulted in normal ear vesicle development (Figure 6C).

### Wnt5a/Ror2 depletion phenocopies the xPAPC morphant phenotype

**Figure 7:**
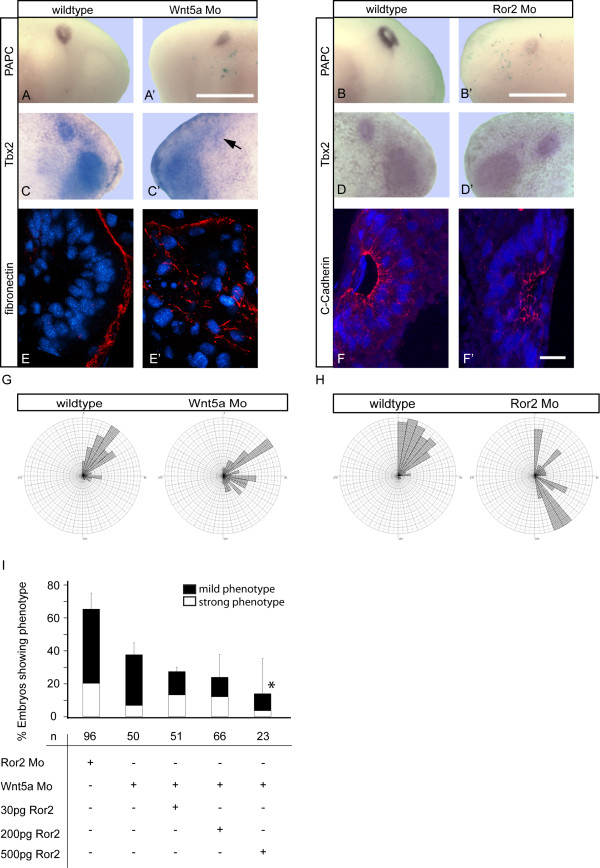
**Knockdown of Wnt5a or Ror2 phenocopies xPAPC depletion**. Knockdown of Wnt5a **(A, A', C, C', E, E') **or Ror2 **(B, B', D, D', F, F')**, respectively, via antisense morpholino injection at 16-cell stage in each of the two adjacent ventral and dorsal cells in the animal pole. **(A, A', B, B', C, C', D, D') **ISH at stage 28 for **(A, A', B, B') **xPAPC or **(C, C', D, D') **Tbx2. Either Wnt5a or Ror2 depletion showed a reduction in xPAPC expression **(A', B') **as well as deformations of the otocyst at the injected side **(A', B', C', D'). (E, E', F, F') **Immunostaining of Wnt5a **(E, E') **or Ror2 **(F, F') **depleted otic epithelia. Mo was injected as described in (A) and ISH for Tbx2 was performed at stage 26. Embryos were then sliced and immunostained for different epithelial markers like **(E, E') **fibronectin or **(F, F') **C-Cadherin in red. The nuclei were stained in blue for DAPI. As in xPAPC depleted embryos the injected otocysts showed epithelial disorganization indicated through inaccurate nuclei alignment and displaced adhesion proteins **(E', F'). (G, H) **Angle measurements of DAPI stained nuclei in immunostained sections reveal a disorganization of the epithelial structure of the otocyst. While in uninjected otocysts DAPI cells are orientated in angles between 0 and 50°mostly, the cells on the injected sides (G: Wnt5a Mo; H: Ror2 Mo) shows angles between 0 and 170°. **(I) **Knockdown of Wnt5a is rescued by Ror2 RNA coinjection in a dose-dependent manner. Injections were carried out at 16-cell stage as described in (A). The embryos were examined at stage 26. Mo, antisense morpholino; n, number of embryos. Scale bar (A', B') 500 μm, (E', F') 20 μm. Arrow points to the deformed otocyst. * = p < 0,05.

Since Wnt5a and Ror2 are also expressed in the inner ear anlage (Figure 1D, F) we investigated their requirement for otic vesicle formation by antisense morpholino injections. The efficiency of the morpholinos has been published previously [18]. Knockdown of Wnt5a but also of Ror2 resulted in a similar phenotype as observed after xPAPC depletion, deformed otocysts and misplaced adhesion molecules. ISH for PAPC showed a reduction in expression (Figure 7A, A', B, B'). ISH for Tbx2 revealed deformed ear vesicles lacking the inner cavity (Figure 7C, C', D, D'). The latter was confirmed by immunostaining of transverse sections with antibodies against fibronectin and C-Cadherin (Figure 7E', F'). Rose diagrams underlined the incorrect columnar alignment of the epithelial cells in the morphants (Figure 7G, H). As shown earlier (Figure 4D) otocyst cells in a wildtype situation are arranged in a columnar orientation with angles between 0 and 60°towards a horizontal median (Figure 7G, H). In case of Wnt5a or Ror2 depletion the orientation of the cell nuclei was strongly affected resulting in a greater variety of angles towards the horizontal median (0 - 170°, Figure 7G, H). In addition knockdown of Wnt5a was rescued by Ror2 RNA injection in a dose dependent manner (Figure 7I). We also studied whether xPAPC or PCNS or a combination of both was able to rescue the otic vesicle formation in Ror2 morphants. This was not observed (data not shown). Therefore, we assume that Wnt5a/Ror2 signaling controls additional unknown factors required in otic vesicle formation. Taken together these results demonstrate that correct apical-basal alignment of the epithelial cells during invagination of placodal tissue depends on xPAPC/RhoA and Wnt5a/Ror2 function.

## Discussion

### xPAPC is required for proper invagination of the otic placode

Building an ear vesicle starting from a thickening ectodermal placode by forming a pit, which closes and separates from the ectoderm, implies multiple changes in cellular behavior. Here we report, that knockdown of xPAPC leads to irregular folding of the otic epithelium and failures in cavity formation. Induction of the otic placode, compartmentalization and neuroblast differentiation was not affected. This is in contrast to an earlier report by Hu et al. [19] who injected dominant-negative forms of xPAPC and showed the complete loss of the otic markers *Tbx2 *and *Sox9 *and an aggregation of pigmented cells instead. This phenotype is not surprising since Chen and Gumbiner [9] demonstrated that expression of M-PAPC similar to overexpression of full-length PAPC led to downregulation in C-Cadherin mediated adhesion. C-Cadherin is the most abundant and ubiquitously expressed cadherin [30] in the early Xenopus embryo. Thus, ectopic expression of M-PAPC might interfere with C-Cadherin in a broad range of tissue formation, which indirectly might affect otic placode formation.

Defects in otocyst formation in the presence of quite normal patterning have been observed in chicken by dominant-negative expression of spalt4/Sall4 mutant [31] or by *Sox9 *knockout in mice [32]. However, instead of an up-folded otic epithelium, loss of these transcription factors resulted in the formation of smaller ear vesicles. Moreover, ectopic expression of spalt4/salI led to super numerous ear vesicle formations, which was not observed by overexpression of xPAPC [19]. Interestingly, both spalt4/sall4 and Sox9 regulate EphA4 expression [31,32]. Moreover, in Sox9 knockout mice, Col2a1 was lost and cell-cell contacts in the otic epithelium were dramatically reduced [32]. Gata3 knockout in mice also resulted in deformed ear vesicles; either they were found small or split into two. Microarray studies displayed a deregulation of EphA4, EphB4, connexin26 (Gjb2) and ostepontin (SSP1) [33]. All these reports on transcription factors point to an important role of cell adhesion regulation during ear vesicle formation. The regulation of PAPC expression seems to be evolutionary conserved. T-box proteins, bHLH proteins of the Mesp family, and Lim1 activate its expression [5,10,11,13,34]. Importantly, activated notch (ICD) and lunatic fringe repress PAPC [10,11]. The inhibitory effect of notch signaling might explain the lack of PAPC expression in the ventrolateral part of the otocyst (see Figure 1B, C), where the neuroblasts delaminate. In chicken lunatic fringe is expressed in this region at corresponding stage [35].

### xPAPC is not primarily required as regulator of cadherin-mediated adhesion in ear vesicle formation

Initially, a direct adhesive function was conferred to PAPC based on homotypic cell sorting experiments [5]. If xPAPC would be required for mediating homophilic cell-adhesion during otic placode invagination, expressing a classical cadherin should restore the weakening of cell adhesion in the morphants. None of the so far tested cadherins was able to rescue vesicle malformation (Figure 5E). Therefore we can exclude that xPAPC is needed to enforce cell-cell adhesion during ear vesicle formation. We also deny a role of PAPC in lowering C-Cadherin mediated adhesion, a mechanism suggested by Chen and Gumbiner [9], because this would suppose that M-PAPC is able to rescue PAPC depletion. This was not observed (Figure 5B). We also could not detect a reduction in E-, C- or XB-Cadherin, β-Catenin or fibronectin immunostaining. Strikingly, the protocadherin PCNS, which is able to rescue PAPC depletion, has been shown to possess little cell-sorting activity in dissociation-reaggregation experiments [27]. The latter indicates that PAPC and PCNS share some other important activities rather than regulating cell adhesion.

### The signaling function of xPAPC and Wnt5a/Ror2 are needed to build up an otocyst

PAPC depletion is rescued by ca RhoA pointing to the signaling function, which is required in otocyst formation. This is in line with the observation that the membrane anchored cytoplasmic domain (C-PAPCgap) shows some rescue ability. The discrepancy in the rescue ability of the full-length form might be explained by the binding of the extracellular domain of PAPC to xFz7 [6]. xFz7 is able to activate β-catenin dependent and independent Wnt-signaling cascades [36]. Interestingly, we could not rescue PAPC depletion in inner ear development by expressing full-length dsh or different dsh constructs, by which PCP/Wnt or β-catenin/Wnt-signaling specifically is activated [28] (summarized in [29]). Knockdown of xFz7 also did not result in an otic vesicle phenotype (data not shown) although the receptor is present in this tissue. We assume that additional members of the Fz-receptor family are expressed and might act redundantly as shown in mice [37].

Wnt5a or Ror2 depletion phenocopied the xPAPC morphant phenotype, a misfolded vesicle and artificially deposited fibronectin and C-Cadherin. Wnt5a and Ror2 morphants showed a reduction but not a complete loss of PAPC in the otic placode visualized by ISH. This could be due to canonical Wnt-signaling, which is present during early inner ear development [20,38]. Apart from Wnt5a/Ror2 PAPC expression is also driven by β-catenin/Wnt signaling [17]. Wnt5a and Ror2 are expressed in the otic vesicle. Although Ror2 and xPAPC morphants showed similarities in the otocyst phenotype xPAPC RNA injections did not rescue the Ror2 morphants. This could rely on the multiple functions of Ror2 in signaling (reviewed in [39]). Ror proteins share with Frizzled receptors the CRD domain which is responsible for Wnt binding. Ror2 has been reported to inhibit canonical Wnt signaling by sequestering Wnt-1 and Wnt-3. However, in other studies Ror2 was shown to promote Wnt-1 activity. Ror2 also influences filopodia formation and cytoskeletal re-arrangements independent of the CRD-domain and Wnt-binding. Thus, Ror2 activity is context dependent. In mouse inner ear development, for example, Ror2 can only activate PCP-signaling in collaboration with the glycoprotein Cthrc1 [40].

In regard to the expression profiles of Wnts, Frizzled-receptors and Wnt-antagonists known from the avian otic primordium [38] a more detailed knowledge of regional differences in the amphibian otocyst is necessary for understanding the Ror2 function. The fate map of mass cell movement in chick otic cup closure [41], instead, asks for an investigation of non-canonical Wnt-signaling in this process.

The similarity in PAPC and Wnt5a/Ror2 depletion phenotypes in Xenopus gastrulation [18] and otocyst formation underlines their conjunction in morphogenetic movements. In this context the signaling function of PAPC in activating the small GTPase RhoA is crucial. As in convergent extension movement [7] and tissue separation [6] xPAPC depletion in the otic placode is rescued by ca RhoA (this paper).

## Conclusions

The protocadherin xPAPC is essential in maintaining proper orientation and alignment of epithelial cells during invagination of the otic placode and cavitation of the otocyst. This morphogentic movement needs xPAPC as activator of RhoA but not as modulator of cadherin mediated cell adhesion. The significance of the signaling part is further supported by the observation that also Wnt5a and Ror2 are required in this morphogenetic process. Furthermore, the non-adhesive PCNS is able to replace xPAPC. How far PCNS can take over all functions of PAPC remains to be answered.

## Methods

### Plasmids, constructs, in vitro transcription

Capped mRNAs were synthesized from linearized templates by using the mMESSAGE mMACHINE kit (Ambion). The antisense probes for in situ hybridization were labeled with digoxigenin by using DIG RNA labeling kit (Roche). C-PAPC and C-PAPCgap in pCS2+ were a kind gift of H. Steinbeisser (Heidelberg).

The used antisense morpholino oligonucleotides (Gene Tools LLC, Philomath, USA) were described previously: PAPC Mo1, PAPC Mo2 [6,7], Wnt5a Mo, Ror2 Mo [18]. The injected Mo amounts are 1.6 pmol XWnt5a Mo and XRor2 Mo. PAPC Morpholinos were injected as a mixture of 1.5 pmol each. The standard control Morpholino was used as negative control. The successful protein synthesis was detected by in vitro transcription and translation kit (TNT^®^, Promega) carried out in accordance with manufacturer's instructions.

### Embryo treatment and in situ hybridization

Eggs were fertilized, cultured and injected as previously described [7]. Embryos were injected into one blastomere at the 2-cell stage or in 2 adjacent cells (ventral and dorsal) at the animal half at 16-cell stage. 4 pg Dextran, 125 pg mRNA encoding for GFP or 40 pg β-Galactosidase RNA were used as lineage tracer. Embryos were fixed in MEMFA (0.1 M MOPS pH 7.2, 2 mM EGTA, 1 mM MgSO_4_, 3.7% formaldehyde). After fixation embryos were stained for β-Galactosidase activity. Whole-mount in situ hybridization was performed according to previously described protocols using the digoxigenin/alkaline phosphatase detection system (Roche) [42].

### Immunocytochemistry

After in situ hybridization embryos were embedded in 2% agarose and sections were obtained by using the Leica VT 1000S vibratome. Sections were blocked for 2 h in 1% BSA/1% horse serum (PAA laboratories GmbH; Invitrogen) in APBST (2.7 mM KCl, 0.15 mM KH_2_PO_4_, 103 mM NaCl, 0.7 mM Na_2_PO_4_, pH 7.5, 0.1% Tween 20). Overnight incubation of the primary antibodies anti-fibronectin (6D9, undiluted supernatant, Developmental Studies Hybridoma Bank), anti-aPKC (1:200, C-20, Santa Cruz Biotechnology) and anti-C-Cadherin (6D6, 1:50, Developmental Studies Hybridoma Bank) was followed by anti-rabbit Cy3 or anti-mouse Cy3 (1:400, Dianova) for 1 h at RT. The injected side was identified by immunostaining with anti-GFP antibody (1:100, Invitrogen).

Quantification of epithelial cell alignment was performed by measuring the angles of the nuclei to the mediolateral axis in transverse sections. For each type of injection 5 embryos with 10 cells per otocyst section were counted. The values were displayed in rose diagrams.

Whole mount immunostaining was performed as described in [43]. The 3A10 monoclonal antibody (developed by Thomas Jessell et al.) was obtained from the Developmental Studies Hybridoma Bank developed under the auspices of the NICHD and maintained by The University of Iowa, Department of Biology, Iowa City, IA 52242.

## Authors' contributions

BJ, AK, AS and DW designed research and DW wrote the manuscript. BJ conducted the research including embryonic experiments and explants. AK performed the cloning of constructs, alignments, and TNT experiments. All authors read and approved the final manuscript.

## Supplementary Material

Additional file 1 **Injection of PAPC Morpholinos has no effect on early induction of the otic placode and on neural differentiation**. **(A, B, C, D, D') **In situ hybridization for Pax8, a marker of the otic placode. At stage 20 the majority of embryos showed no phenotype **(A) **while some showed a reduction in Pax8 expression at the PAPC Mo injected side **(B)**. At stage 22 **(C) **or 24 **(D, D') **no significant alterations were observed upon PAPC depletion. **(E, E', G, G') **Expression of neural markers like Tbx 1 **(E, E')**, RunX1 **(G, G') **was not significantly affected by PAPC Mo injections. **(F) **The peripheral nervous system appeared normal when immunostained with the neurofilament antibody 3A10. **(H) **Statistical analysis of embryos showing a reduced marker expression by PAPC morpholino treatment. A statistical significant increase in embryos with decreased expression was only observed in stage 22 embryos probed with Pax8. Asterix marks the injected side (A-G'). Scale bar 250 μm. Click here for file
