# Supplementary figures and images for: PAPC and the Wnt5a/Ror2 pathway control the invagination of the otic placode in Xenopus
Source: BMC Dev Biol. 2011 Jun 10;11:36. doi: 10.1186/1471-213X-11-36 (PMC3127988; doi:10.1186/1471-213X-11-36)

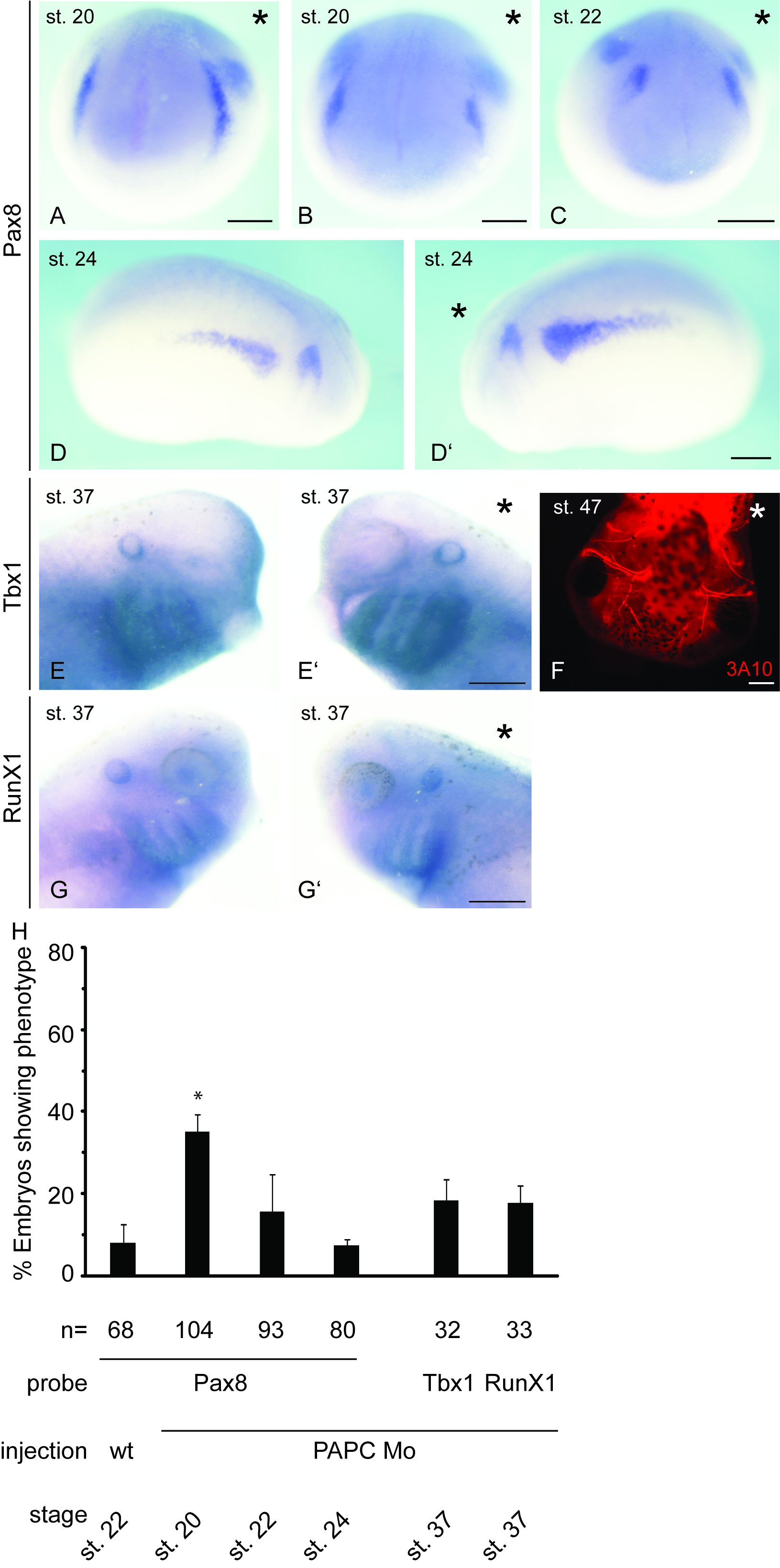

Supplement: Additional file 1 — Injection of PAPC Morpholinos has no effect on early induction of the otic placode and on neural differentiation. (A, B, C, D, D') In situ hybridization for Pax8, a marker of the otic placode. At stage 20 the majority of embryos showed no phenotype (A) while some showed a reduction in Pax8 expression at the PAPC Mo injected side (B). At stage 22 (C) or 24 (D, D') no significant alterations were observed upon PAPC depletion. (E, E', G, G') Expression of neural markers like Tbx 1 (E, E'), RunX1 (G, G') was not significantly affected by PAPC Mo injections. (F) The peripheral nervous system appeared normal when immunostained with the neurofilament antibody 3A10. (H) Statistical analysis of embryos showing a reduced marker expression by PAPC morpholino treatment. A statistical significant increase in embryos with decreased expression was only observed in stage 22 embryos probed with Pax8. Asterix marks the injected side (A-G'). Scale bar 250 μm. [file 1471-213X-11-36-S1.TIFF]
